# Alcohol Consumption, Genetic Variants in Alcohol Deydrogenases, and Risk of Cardiovascular Diseases: A Prospective Study and Meta-Analysis

**DOI:** 10.1371/journal.pone.0032176

**Published:** 2012-02-21

**Authors:** Dagmar Drogan, Abigail J. Sheldrick, Madlen Schütze, Sven Knüppel, Frank Andersohn, Romina di Giuseppe, Bianca Herrmann, Stefan N. Willich, Edeltraut Garbe, Manuela M. Bergmann, Heiner Boeing, Cornelia Weikert

**Affiliations:** 1 Department of Epidemiology, German Institute of Human Nutrition Potsdam-Rehbruecke, Nuthetal, Germany; 2 Institute of Clinical Pharmacology and Toxicology; Charité University Medical Center, Berlin, Germany; 3 Institute for Social Medicine, Epidemiology and Health Economics, Charité University Medical Center, Berlin, Germany; 4 Department of Clinical Epidemiology, Bremen Institute for Prevention Research and Social Medicine, Bremen, Germany; 5 Department of Psychiatry, Psychotherapy and Psychosomatics, Medical School, RWTH Aachen University, Aachen, Germany; 6 Department 4.1 Biology in Materials Protection and Environmental Issues, Federal Institute for Materials Research and Testing, Berlin, Germany; Chinese Academy of Medical Sciences, China

## Abstract

**Objective:**

First, to investigate and compare associations between alcohol consumption and variants in alcohol dehydrogenase (ADH) genes with incidence of cardiovascular diseases (CVD) in a large German cohort. Second, to quantitatively summarize available evidence of prospective studies on polymorphisms in *ADH1B* and *ADH1C* and CVD-risk.

**Methods:**

We conducted a case-cohort study nested within the European Prospective Investigation into Cancer and Nutrition (EPIC)-Potsdam cohort including a randomly drawn subcohort (n = 2175) and incident cases of myocardial infarction (MI; n = 230) or stroke (n = 208). Mean follow-up time was 8.2±2.2 years. The association between alcohol consumption, *ADH1B* or *ADH1C* genotypes, and CVD-risk was assessed using Cox proportional hazards regression. Additionally, we report results on associations of variants in *ADH1B* and *ADH1C* with ischemic heart disease and stroke in the context of a meta-analysis of previously published prospective studies published up to November 2011.

**Results:**

Compared to individuals who drank >0 to 6 g alcohol/d, we observed a reduced risk of MI among females consuming >12 g alcohol/d (HR = 0.31; 95% CI: 0.10–0.97) and among males consuming >24 to 60 g/d (HR = 0.57; 95% CI: 0.33–0.98) or >60 g alcohol/d (HR = 0.30; 95% CI: 0.12–0.78). Stroke risk was not significantly related to alcohol consumption >6 g/d, but we observed an increased risk of stroke in men reporting no alcohol consumption. Individuals with the slow-coding ADH1B*1/1 genotype reported higher median alcohol consumption. Yet, polymorphisms in *ADH1B* or *ADH1C* were not significantly associated with risk of CVD in our data and after pooling results of eligible prospective studies [ADH1B*1/1: RR = 1.35 (95% CI: 0.98–1.88; p for heterogeneity: 0.364); ADH1C*2/2: RR = 1.07 (95% CI: 0.90–1.27; p for heterogeneity: 0.098)].

**Conclusion:**

The well described association between alcohol consumption and CVD-risk is not reflected by ADH polymorphisms, which modify the rate of ethanol oxidation.

## Introduction

Considerable epidemiologic evidence links moderate alcohol consumption to a reduced risk of coronary heart disease (CHD) and stroke [1]. Because alcohol consumption is related to many potentially confounding factors, the causal effect of alcohol consumption on CVD risk is difficult to assess in observational studies. Long-term randomized controlled trials investigating the effects of regular alcohol consumption on hard cardiovascular endpoints are unlikely to be ever carried out. However, short- to medium-term intervention studies have demonstrated a favorable effect of ethanol on lipids, haemostasis, and insulin sensitivity [2]. As biologically plausible mediators these mechanisms may be viewed as an indirect support for a protective effect of moderate alcohol use on cardiovascular events.

In addition, genetic variants associated with average alcohol consumption and/or circulating ethanol levels may act as unconfounded and precisely measured markers of the exposure and thereby contributing to strengthen causal inference [3]. In this respect, genes coding for alcohol dehydrogenases (ADH) deserve attention. ADHs catalyze the oxidation of alcohols to aldehydes. In humans, five ADH classes exist and functional polymorphisms in the genes *ADH1B* and *ADH1C* produce isoenzymes with different maximal activities (V_max_) and affinities for ethanol [4]. At the *ADH1C* locus, two single nucleotide polymorphisms (SNPs) in very strong linkage disequilibrium have been described [5]. The respective alleles ADH1C*1 and ADH1C*2 code for two ADH subunits (g1 and g2) which differ at amino acid positions 271 and 349. Compared to the homodimeric g2 isoenzyme, g1g1 has a 2.5-fold higher V_max_ of ethanol oxidation [6]. An even 40-fold difference in V_max_ has been observed between b1b1 and the fast metabolizing b2b2 isoenzyme, resulting from variation at rs1229984 (*ADH1B* Arg48His) [4].

A few prospective studies have investigated the association between alcohol consumption and risk of cardiovascular diseases (CVD) in combination with variations in genes coding for ADH1C or ADH1B [7], [8], [9], [10], [11], [12], [13]. In line with the hypothesis that ethanol is largely responsible for the inverse association between alcohol consumption and coronary events, individuals with the slow ADH1C*2/2 genotype had a lower risk of myocardial infarction (MI) in a study among male physicians [9]. However, later studies on this subject were inconsistent [7], [8], [10], [11], [12]. Comparable data on polymorphisms in *ADH1B* is rare because of the low frequency of the ADH1B^*^2 allele in caucasians [14]. Risk of MI did not change across *ADH1B* genotypes in a Danish population [10], whereas the ADH1B*1 allele coding for the slow isoenzyme was associated with a higher prevalence of cerebral infarction in Japanese men but not in women [15].

In this study we examined and compared the associations of alcohol consumption and SNPs in *ADH1C* and *ADH1B* with incident cardiovascular events in a prospective cohort. To summarize the available evidence on polymorphisms in the above genes with ischemic heart disease and stroke, we also carried out a meta-analysis of prospective population-based studies.

## Methods

### Ethics statement

Study procedures were approved by the Ethics Committee of the medical association of the State of Brandenburg (Germany) and all participants gave their written informed consent.

### Study design and study population

The European Prospective Investigation into Cancer and Nutrition (EPIC)-Potsdam study is part of the large-scale EPIC cohort and includes 10,904 male and 16,644 female participants recruited from the general population of Potsdam and surrounding areas. The preferred age range for recruitment was 35–65 years. Baseline examination was conducted from 1994 through 1998 and included blood sampling, measurements of blood pressure and anthropometric parameters, self-administered questionnaires on diet and lifestyle, and personal computer-assisted interviews [16].

Data on *ADH1C* and *ADH1B* genotypes was not available for the whole cohort, but only for a case-cohort study nested within the EPIC-Potsdam cohort, which has been described elsewhere [17]. With this type of study design, the results are expected to be generalizable to the source population without the need to measure the polymorphisms in the entire cohort [18]. For reasons of consistency, all analyses were performed in this case-cohort study.

#### Selection of cases

After excluding EPIC-Potsdam participants with a history of MI or stroke at baseline, we identified 269 individuals with incident MI and 246 individuals with incident stroke occurring between baseline examination and 30^th^ November 2006 (mean follow-up time 8.2±2.2 years). In 6 individuals with more than one cardiovascular event, only the first event was considered. For the present analyses, we excluded 41 cases of MI and 34 stroke cases because blood specimens were not available or because of missing values in relevant covariates, dietary or genetic data.

#### Selection of the subcohort

For the subcohort, we randomly selected 2500 individuals from the EPIC-Potsdam cohort. Consistent with the applied case-cohort design, the subcohort included 60 subjects who experienced MI or stroke during the study period. In the subcohort, we excluded participants with a history of MI or stroke at baseline, with missing follow-up data, unavailable blood samples, or missing information on relevant covariates, alcohol consumption data or the SNPs.

Thus, our analyses are based on a case-cohort sample of 2558 individuals (230 MI cases, 208 stroke cases, and 2175 who remained free of MI or stroke during follow-up).

### Ascertainment of incident MI and stroke

Potential cases of incident MI or stroke were identified by death certificate or by self-report in one of the four biennial follow-up questionnaires [19]. To reduce the number of false-negative reports, the questionnaire also contained items on stroke symptoms, as described earlier [20]. The diagnosis was verified by review of medical records from the hospital, by contacting the patients' attending physician, or by review of death certificates according to WHO criteria. Cases were subsequently classified as incident MI (ICD-10 I21), ischemic stroke (ICD-10 I63.0–I63.9), intracerebral (ICD-10 I61.0–I61.9) or subarachnoidal hemorrhage (ICD-10 I60.0–I60.9), or undetermined stroke (ICD-10 I64.0–I64.9) [21].

### Assessment of alcohol consumption

Dietary habits including alcohol consumption were assessed at baseline by use of a validated, self-administered food frequency questionnaire [22]. Baseline alcohol consumption was calculated based on the reported number of glasses of alcoholic beverages consumed during the 12 months prior to recruitment. Assuming one standard drink to be equivalent to 12 g of pure alcohol [23], baseline alcohol consumption was modeled using the following categories: non-drinker, >0 to 6 g/d (>0 to 0.5 drink/d), >6 to 12 g/d (>0.5 to 1 drink/d), >12 to 24 g/d (>1 to 2 drinks/d), >24 to 60 g/d (>2 to 5 drinks/d), >60 g/d (>5 drinks/d). Because the average alcohol consumption was lower among the women than among the men, we combined the two highest consumption categories in women. Information on past consumption of alcoholic beverages at 20, 30, and 40 years of age was obtained by a lifestyle questionnaire. This information was combined to define three groups: never use of alcohol in the past, heavy alcohol consumption at one or more time points in the past, never heavy alcohol consumption at any time in the past. Heavy alcohol consumption was defined as 60 g/d in men and 30 g/d in women, thus reflecting 2.5 times the upper recommended limit of two standard drinks a day for men and one standard drink a day for women [24].

### Assessment of covariates

Smoking habits, physical activity, educational attainment, and medical history were assessed during a standardized interview. We divided the study population according to their smoking status into never smokers, former smokers >5 years, former smokers ≤5 years, smokers <20 units/day, and smokers ≥20 units/day; with one unit being equivalent to one cigarette/cigar/pipe. Physical activity was expressed as the mean duration of leisure time physical activities during the summer and winter seasons. This variable was dichotomized using a cut point of ≥2 h/week. Educational attainment was expressed as vocational school or less, technical school, and university degree.

Baseline measurements of anthropometric parameters and blood pressure were obtained by trained personnel [25]. BMI was calculated as the ratio of body weight (kg) to height squared (m^2^). Systolic and diastolic blood pressure (BP) was measured after a resting period of 15–30 minutes. Prevalent hypertension was defined as systolic BP≥140 mmHg, diastolic BP≥90 mmHg, self-reported hypertension diagnosis, or use of antihypertensive medication. The prevalence of diabetes mellitus at baseline was evaluated by a study physician using information on self-reported medical diagnosis, medication records, and dieting behaviors. In ambiguous cases, the diagnosis was confirmed by personal communication with the participant and/or treating physician.

### Blood collection and laboratory analyses

During baseline examination, peripheral venous citrate blood samples were taken and subsequently centrifuged at 1000× g for 10 min at 4°C. Plasma was aliquoted and stored in liquid nitrogen at −196°C. Plasma levels of total cholesterol were measured with the automatic ADVIA 1650 analyzer (Siemens Medical Solutions, Erlangen, Germany). Genomic DNA was extracted from peripheral blood leukocytes using the Qiagen QIAamp 96 Blood Kit. Following whole genome amplification (GenomiPhi DNA Amplification Kit, Amnersham Biosciences), genotyping was performed with the TaqMan SNP Genotyping Assay using the ABI PRISM 7900HT Sequence Detection System according to the manufacturer's protocol (Applied Biosystems, Foster City, CA, USA). The genotyping success rates in SNPs rs1229984 and rs698 were 98.8% and 99.4%, respectively.

### Statistical analysis

Statistical analysis was performed using SAS software package, release 9.2 (SAS Institute, Cary, NC). All tests were performed two-sided with p<0.05 considered as statistically significant.

For subjects of the subcohort we calculated means ± standard deviation (SD) or frequencies of selected baseline characteristics across categories of alcohol consumption. As drinking habits and participant characteristics are likely to differ between men and women, gender-specific values are presented. To investigate the association of alcohol consumption with risk of CVD, MI, and stroke, we conducted Cox proportional hazards regression adapted for the case-cohort design using the weighting method described by Prentice [18]. To examine the association of alcohol consumption with CVD risk, gender-specific multivariable-adjusted hazard ratios (HR) were derived using subjects with an alcohol consumption of >0 to 6 g/d as the reference group. Because of the dependence among observations introduced by the case-cohort design, we used robust standard errors obtained from the robust sandwich covariance estimates for calculating 95% confidence intervals (CI). For the counting process style of input, the subjects' age at recruitment was used as the entry time and age at censoring or at diagnosis of MI or stroke as the exit time. All models were stratified according to age at recruitment in one year categories and adjusted for BMI (kg/m^2^), waist circumference (cm), smoking status (never smokers, former smokers >5 years, former smokers ≤5 years, smokers <20 units/day, and smokers ≥20 units/day), educational attainment (vocational school or less, technical school, university), physical activity ≥2 h/week, non-alcohol energy intake (kJ/d), prevalent hypertension, prevalent diabetes mellitus, and plasma total cholesterol level. In additional models we also adjusted for reported past alcohol consumption and polymorphisms in *ADH1B* and *ADH1C*.

The χ^2^ test was used to determine whether the distributions of *ADH1B* and *ADH1C* genotypes were in Hardy-Weinberg equilibrium among subjects of the subcohort. As expected from their random allocation at meiosis, the distribution of the investigated alleles was comparable between men and women. Therefore, to increase statistical power, both genders were combined for all analyses involving SNP data. We also examined the associations between ADH genotypes with risk of MI/stroke in models stratified by age and adjusting for the above mentioned covariates plus gender. For that purpose, subjects with a genotype coding for a faster isoenzyme – ADH1C*1/1 and ADH1B*1/2 or *2/2* – were used as the respective reference group. The impact of alcohol consumption on these associations was analyzed in models additionally adjusted for the categories of baseline alcohol consumption defined above. To test for interaction, we used a likelihood ratio test to compare a model including alcohol consumption (0 g/d, >0 to 12 g/d (women)/>0 to 24 g/d (men), >12 g/d (women)/>24 g/d (men)) and the *ADH1C* genotypes with a model additionally including the interaction terms. Because of low number of cases with the fast-metabolizing ADH1B*1/2 or *2/2* genotypes, we were unable to further stratify this group and therefore, no p-values for interactions are reported for *ADH1B*.

The power for detecting an association between *ADH* polymorphisms and MI or stroke was calculated based on an algorithm developed for case-cohort studies [26] and assuming an incidence rate of 0.01, a sampling rate of 0.08 for the random subcohort, and an allele frequency of 58% for the ADH1C*1 allele and 2% for the *ADH1B^.^2* allele [27]. With an alpha of 0.05, we had 84% power to detect an HR for either MI or stroke of 0.70 for the comparison of ADH1C*2/2 (slow) versus ADH1C*1/1 (fast), which is an effect size similar to what was found by Hines et al. [9]. For the comparison of individuals with the ADH1B^*^1/1 genotype (slow) with ADH1B*1/2 or ADH1B*2/2 (fast), power was only 43% to detect an HR of 0.70. That's why we also conducted a meta-analysis to pool our results with results obtained from comparable studies.

### Meta-analysis

For a meta-analysis, we aimed to identify prospective studies investigating SNPs in the *ADH1B* or *ADH1C* gene in relation to incidence of ischemic heart disease or stroke. For that purpose we searched Medline, EMBASE, and Web of Science for studies published by November 24, 2011. No language restriction was applied. The following search strategy was applied: 1) Coronary OR Myocardial OR Heart OR Cerebral OR Cerebrovascular OR Brain; 2) Syndrome OR Disease* OR Infarction* OR Attack*; 3) (#1 AND #2); 4) Alcohol dehydrogenase*; 5) Prospective OR Cohort OR Longitudinal OR Incidence; 6) #3 AND #4 AND #5. The search in Medline and EMBASE was limited to human studies. For the Medline search, we additionally included the terms “OR „Myocardial Ischemia“ [MeSH] OR „Stroke“ [MeSH]” in #3, “OR „Alcohol Dehydrogenase“ [MeSH]” in #4, “OR „Cohort studies“ [MeSH]” in #5. In EMBASE, we replaced these MeSH terms by the respective index terms ‘Ischemic Heart Disease’, ‘Stroke’, ‘Alcohol Dehydrogenase’, and ‘Cohort Studies’.

Our search identified 44 references which were reviewed by two independent assessors (DD and RdG). A total of 36 references were excluded for the following reasons: no original data (n = 15), no relevant outcome (n = 16), no relevant exposure (n = 3), no relevant study design (n = 2). Thus, eight studies met our inclusion criteria of which one was examining stroke [13]. Eligible studies on ischemic heart disease included studies on CHD [7], [8], [12], MI [9], [10], and acute coronary events [11]. In total, two of the identified studies analyzed SNP rs1229984 in the *ADH1B* gene [10], [11] and seven analyzed either SNP rs698 [8], [9], [10], [12], [13] or rs1693482 [7], [11] in the *ADH1C* gene. Because the latter are in very high linkage disequilibrium [5], we combined this data. Scanning the references of the retrieved reports did not lead to as yet unknown eligible studies.

Two authors (DD and RdG) independently extracted and tabulated information on study design, study population, sample size, the number of cases, outcome, the investigated SNP as well as point estimates and 95% CI from the original reports. Discrepancies were resolved via review of the original articles. For three studies that did not provide information on risk estimates and 95% CI, we attempted to obtain it by correspondence with the authors [7], [11], [12]:

- One reply enabled additional unpublished data of the Second Northwick Park Heart to be incorporated in the meta-analysis (PJ Talmud, personal communication) [12].
- One study was excluded from the meta-analysis because we were unable to obtain the requested results [11].
- Because we were unable to obtain data for the study of Ebrahim et al. [7], we used the reported crude incidence rates of CHD across *ADH1C* genotypes to calculate Relative Risks (RR) and 95% CI as described by Rothman et al. [28].

The meta-analysis was performed using the R software (version 2.12.1) and the package meta [29]. Pooled estimates of RR and 95% CI were obtained by means of a random effects approach with study-specific exposure-disease effects weighted according to the inverse of their variances plus the common between-studies variance [30]. Heterogeneity was estimated using the I^2^ statistic and tested using the Q statistic. Publication bias was evaluated by visual inspection of the funnel plot and the tests of Begg and Mazumdar [31] and of Egger et al. [32]. These tests were conducted for data on ADH1C only, because only one report was eligible for the meta-analysis on ADH1B in addition to our own data.

## Results

The present analyses are based on 2558 subjects including 438 major CVD events (MI: n = 230; IS: n = 169; HS: n = 35; undetermined stroke: n = 4). Among subcohort participants about 50% of women consumed >0 to 6 g/d (Table 1) whereas most men (32%) reported an alcohol consumption of >24 to 60 g/d (Table 2). In both genders, never smoking was most prevalent in those reporting low alcohol consumption (>0 to 6 g/d) and the highest proportion of prevalent type 2 diabetes was observed among individuals with zero alcohol consumption at baseline.

**Table 1:** Baseline characteristics across drinking categories in 1360 women of a randomly drawn subcohort.

Alcohol consumption	0 g/dn = 35 (2.6%)	>0 to 6 g/dn = 708 (58.1%)	>6 to 12 g/dn = 324 (23.8%)	>12 to 24 g/dn = 192 (14.1%)	>24 g/dn = 101 (7.4%)
Age, y, mean ± SD	52.6±11.5	49.6±9.4	47.9±9.0	47.8±8.8	47.3±8.6
BMI, kg/m^2^, mean ± SD	25.5±4.8	26.1±4.9	25.5±4.6	25.2±4.1	25±4.0
Waist circumference, cm, mean ± SD	80.5±11.5	81.5±12.3	80.2±11.3	79.8±10.8	79.1±10.5
*ADH1C* genotype, %					
ADH1C*1/1 (fast)	28.6	34.6	32.7	39.1	29.7
ADH1C*1/2 (intermediate)	54.3	48.7	50.0	47.4	51.5
ADH1C*2/2 (slow)	17.1	16.7	17.3	13.5	18.8
ADH1B*1/1 (slow)	88.6	90.7	92	92.2	92.1
Smoking status, %					
Never smokers	57.1	63.6	56.5	48.4	39.6
Past smokers >5 years	17.1	13.6	18.8	24.5	26.7
Past smokers ≤5 years	5.7	6.6	8.6	5.2	8.9
Current smokers <20 units	14.3	13.6	13.6	16.1	19.8
Current smokers ≥20 units	5.7	2.7	2.5	5.7	5.0
University degree, %	37.1	23.2	36.1	38.5	31.7
Physical activity > = 2 h/week, %	31.4	21.9	24.4	26.6	29.7
Hypertension, %	57.1	41.9	40.1	37.0	42.6
Diabetes mellitus, %	5.7	3.5	0.6	3.6	5.0
Total cholesterol, mg/dl, mean ± SD	182.5±43.0	174.1±36.3	166.2±40.7	171.1±38.0	171.4±35.9

**Table 2:** Baseline characteristics across categories of baseline alcohol consumption in 815 men of a randomly drawn subcohort.

Alcohol consumption	0 g/dn = 26 (43.3%)	>0 to 6 g/dn = 137 (16.8%)	>6 to 12 g/dn = 135 (16.6%)	>12 to 24 g/dn = 200 (24.5%)	>24 to 60 g/dn = 257 (31.5%)	>60 g/dn = 60 (7.4%)
Age, y, mean ± SD	49.7±8.6	52.8±8.3	53.0±7.9	51.7±8.2	51.4±8.0	53.0±8.1
BMI, kg/m^2^, mean ± SD	25.3±3.9	26.6±4.0	27.1±3.7	26.7±3.4	26.5±3.1	26.9±4.0
Waist circumference, cm, mean ± SD	90.5±11.5	92.9±9.9	94.7±10.2	93.9±9.7	93.7±9.3	95.6±12.1
*ADH1C* genotype, %						
ADH1C*1/1 (fast)	34.6	32.8	39.3	35.0	34.2	31.7
ADH1C*1/2 (intermediate)	46.2	48.2	41.5	46.0	48.2	46.7
ADH1C*2/2 (slow)	19.2	19.0	19.3	19.0	17.5	21.7
ADH1B*1/1 (slow)	96.2	83.9	92.6	94.0	93.4	98.3
Smoking status, %						
Never smokers	7.7	35.8	34.1	33.5	25.7	16.7
Past smokers >5 years	23.1	32.1	26.7	37.0	39.7	38.3
Past smokers ≤5 years	3.8	8.0	13.3	7.5	8.2	6.7
Current smokers <20 units	26.9	12.4	17.0	16.0	16.7	20.0
Current smokers ≥20 units	38.5	11.7	8.9	6.0	9.7	18.3
University degree, %	38.5	46.0	57.8	52.5	61.9	41.7
Physical activity ≥2 h/week, %	15.4	26.3	22.2	22.0	26.8	23.3
Hypertension, %	53.8	49.6	63.0	60.5	57.2	63.3
Diabetes mellitus, %	15.4	5.8	7.4	4.5	3.5	10.0
Total cholesterol, mg/dl, mean ± SD	171.4±55.3	170.2±31.4	175.8±37.0	178.6±33.1	179.5±41.3	186.3±36.7

Compared to individuals consuming >0 to 6 g alcohol/d there was a significantly lower risk of MI among females consuming >12 to 24 g alcohol/d (HR = 0.31; 95% CI 0.10 to 0.97; Table 3) and among males consuming >24 to 60 g/d (HR = 0.57; 95% CI 0.33 to 0.98) or >60 g/d (HR = 0.30; 95% CI 0.12 to 0.78; Table 4). Further adjustment for past drinking behaviors or genotypes of *ADH1B* and *ADH1C* did not materially change risk estimates associated with moderate to high alcohol consumption. Because only one man and 14 women reported lifelong abstinence from alcohol, we had insufficient power to derive risk estimates for this group. Excluding them from the analyses resulted in a HR = 1.51 (95% CI: 0.62–3.68) for men and a HR = 1.52 (95% CI: 0.41–5.61) for women consuming no alcoholic beverages at baseline but with an alcohol consumption>0 g/d at any point in their past.

**Table 3:** Relative Risks of MI and stroke across categories of baseline alcohol consumption in women.

Outcome	0 g/d	>0 to 6 g/d	>6 to 12 g/d	>12 to 24 g/d	>24 g/d
**MI**					
Cases (n)	5	40	12	4	0
Person-Years	285	5966	2735	1553	805
HR (95% CI)					
Model 1	0.99 (0.30, 3.23)	1 (Ref)	0.74 (0.36, 1.53)	0.31 (0.10, 0.97)	n.a.
Model 2	0.72 (0.19, 2.81)	1 (Ref)	0.75 (0.36, 1.55)	0.30 (0.10, 0.91)	n.a.
Model 3	1.14 (0.35, 3.73)	1 (Ref)	0.69 (0.32, 1.48)	0.32 (0.10, 1.03)	n.a.
**Stroke**					
Cases (n)	6	47	23	12	7
Person-Years	295	5983	2757	1582	835
HR (95% CI)					
Model 1	1.70 (0.66, 4.40)	1 (Ref)	1.26 (0.73, 2.17)	1.00 (0.51, 1.98)	1.05 (0.43, 2.58)
Model 2	1.32 (0.46, 3.81)	1 (Ref)	1.27 (0.73, 2.22)	0.98 (0.50, 1.92)	0.87 (0.35, 2.19)
Model 3	1.71 (0.67, 4.38)	1 (Ref)	1.24 (0.71, 2.13)	1.00 (0.50, 1.99)	1.03 (0.41, 2.56)

Model 1 stratified by age at recruitment and adjusted for BMI, waist circumference, smoking status, educational attainment, physical activity, non-alcohol energy intake, prevalent hypertension, prevalent diabetes mellitus, and plasma total cholesterol level.

Model 2: Model 1 plus past alcohol consumption.

Model 3: Model 1 plus *ADH1C* and *ADH1B* genotypes.

**Table 4:** Relative Risks of MI and stroke across categories of baseline alcohol consumption in men.

Outcome	0 g/d	>0 to 6 g/d	>6 to 12 g/d	>12 to 24 g/d	>24 to 60 g/d	>60 g/d
**MI**						
Cases (n)	11	39	35	32	45	7
Person-Years	226	1231	1247	1755	2227	508
HR (95% CI)						
Model 1	1.48 (0.61, 3.58)	1 (Ref)	0.76 (0.44, 1.33)	0.56 (0.32, 1.00)	0.57 (0.33, 0.98)	0.30 (0.12, 0.78)
Model 2	1.38 (0.54, 3.55)	1 (Ref)	0.76 (0.44, 1.33)	0.57 (0.32, 1.00)	0.56 (0.32, 0.97)	0.27 (0.10, 0.74)
Model 3	1.37 (0.57, 3.28)	1 (Ref)	0.69 (0.39, 1.22)	0.51 (0.29, 0.91)	0.53 (0.31, 0.90)	0.27 (0.10, 0.71)
**Stroke**						
Cases (n)	6	18	18	24	36	11
Person-Years	215	1144	1174	1747	2185	520
HR (95% CI)						
Model 1	3.57 (1.13, 11.32)	1 (Ref)	0.98 (0.47, 2.02)	1.13 (0.57, 2.24)	1.57 (0.82, 3.00)	1.50 (0.67, 3.36)
Model 2	3.69 (1.07, 12.69)	1 (Ref)	0.98 (0.47, 2.03)	1.13 (0.57, 2.24)	1.59 (0.83, 3.04)	1.53 (0.67, 3.50)
Model 3	3.48 (1.10, 10.95)	1 (Ref)	0.94 (0.45, 1.94)	1.11 (0.56, 2.20)	1.54 (0.80, 2.95)	1.46 (0.65, 3.28)

Model 1 stratified by age at recruitment and adjusted for BMI, waist circumference, smoking status, educational attainment, physical activity, non-alcohol energy intake, prevalent hypertension, prevalent diabetes mellitus, and plasma total cholesterol level.

Model 2: Model 1 plus past alcohol consumption.

Model 3: Model 1 plus *ADH1C* and *ADH1B* genotypes.

Risk of stroke was not significantly related to alcohol consumption above 6 g/d (Tables 3 and 4). However, abstinence from alcohol at baseline was associated with a significantly increased risk of stroke in men (HR = 3.57; 95% CI: 1.13–11.32). We had insufficient case numbers to perform a sub-analysis for hemorrhagic stroke. Yet, when restricting the outcome to ischemic stroke the HR for >6 to12 g/d, >12 to 24 g/d, >24 to 60 g/d, and >60 g/d were 1.01 (95% CI: 0.47–2.17), 1.07 (95% CI: 0.51–2.25), 1.68 (95% CI: 0.85–3.32), 1.01 (95% CI: 0.41–2.46) in men compared to the reference group (data not shown in a table). In women, HR of ischemic stroke associated with >6 to12 g/d, >12 to 24 g/d, >24 g/d were 1.28 (95% CI: 0.71–2.31), 1.11 (95% CI: 0.53–2.30), and 0.52 (95% CI: 0.14–1.87), respectively.

**Figure 1:**
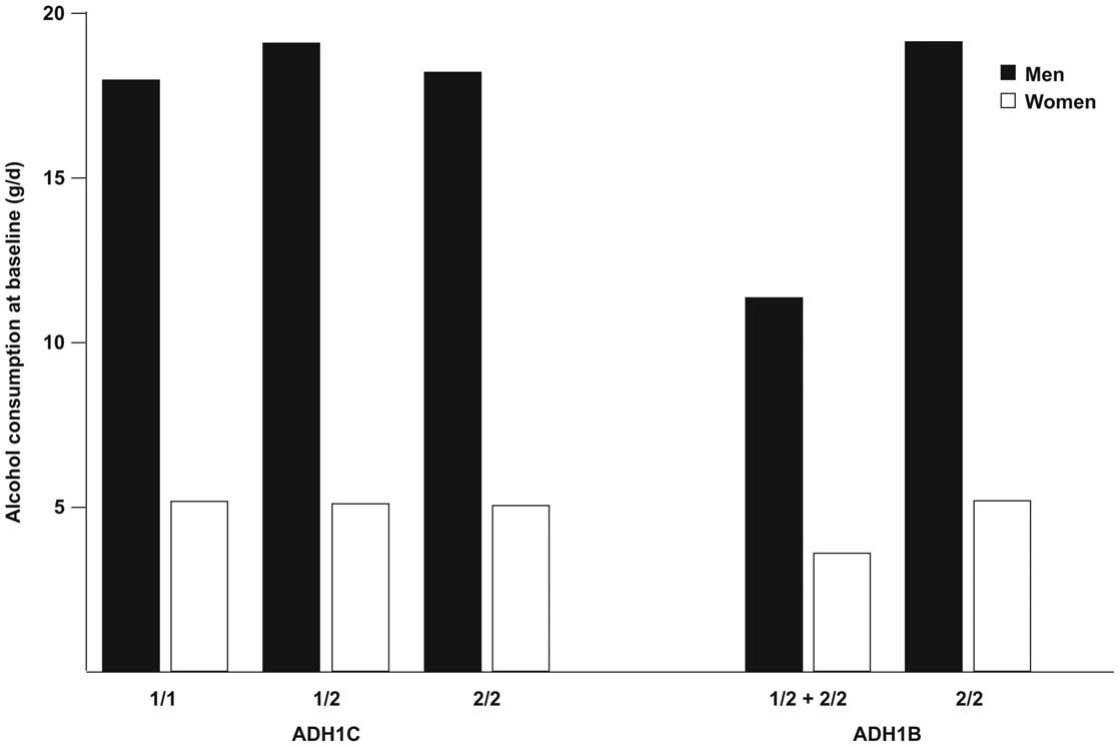
Median baseline alcohol consumption across genotypes of *ADH1C* and *ADH1B*. Data derived from 815 men and 1360 women of a randomly drawn subcohort of the EPIC-Potsdam study.

Among the individuals of the subcohort, frequencies of ADH1B*1/1 and ADH1C*2/2 coding for the respective less active isoenzymes were 91.6% and 17.4%. The distributions of the investigated *ADH* genotypes were in Hardy-Weinberg equilibrium (*ADH1B*: p = 0.991; *ADH1C*: p = 0.993). We observed no significant association between *ADH1C* genotypes and alcohol consumption (Tables 1 and 2, Figure 1). In comparison, the frequency of the slow metabolizing *ADH1B* variant (ADH1B*1/1) was lowest in men consuming >0 to 6 g/d and gradually increased with increasing alcohol consumption (Table 1). In line with this observation, median alcohol consumption was 7.8 g/d higher in males carrying the ADH1B*1/1 genotype as compared to males with the ADH1B*1/2 or ADH1B*2/2 genotype, respectively (Figure 1; p = 0.001). The same tendency was observed in women, but the differences in alcohol consumption were very low (1.6 g/d; p = 0.046).

**Figure 2:**
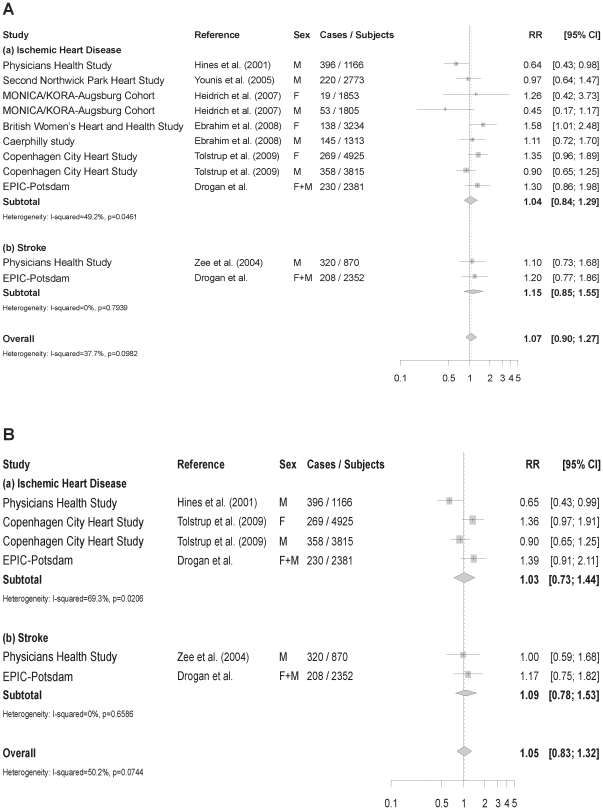
Relative Risks and 95% CI from a meta-analysis of rs698 or rs1693482 in the *ADH1C* gene and CVD. Estimates are for the comparison of the slow-coding genotype (ADH1C*2/2) with the fast-coding genotype (ADH1C*1/1) (A) Including risk estimates not adjusted for alcohol consumption. (B) Including risk estimates adjusted for alcohol consumption. Data for the Second Northwick Park Heart Study obtained by personal communication with the corresponding author.

**Figure 3:**
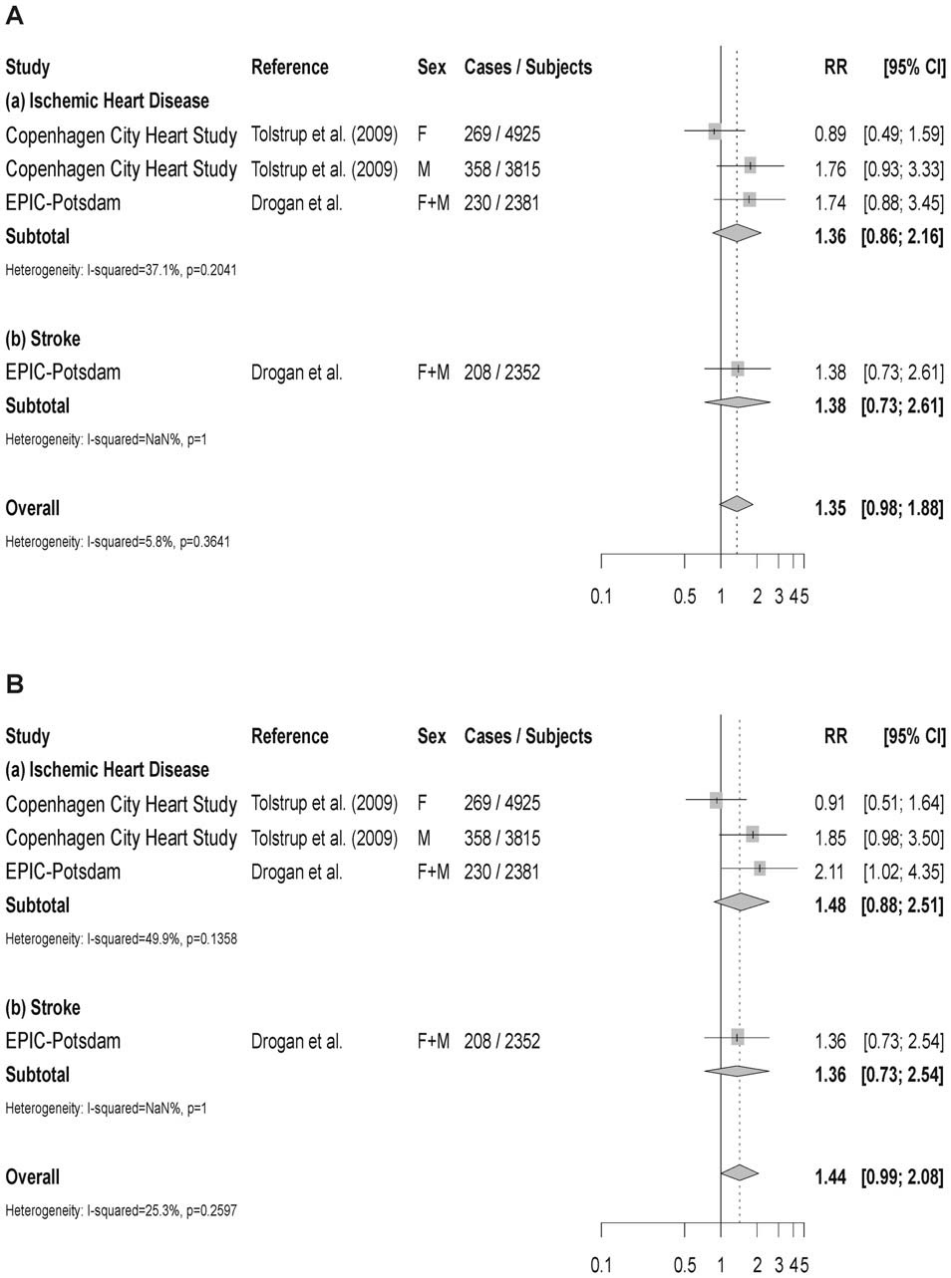
Relative Risks and 95% CI of CVD from a meta-analysis of rs1229984 in the *ADH1B* gene and CVD. Estimates are for the comparison of the slow-coding genotype (ADH1B*1/1) with the fast-coding genotype (ADH1B*1/2 or *2/2*). (A) Including risk estimates not adjusted for alcohol consumption. (B) Including risk estimates adjusted for alcohol consumption.

In multivariable-adjusted models *ADH1C* and *ADH1B* genotypes were not significantly associated with risk of MI or stroke in our cohort (Figures 2A and 3A). Further adjustment for baseline alcohol consumption only slightly changed risk estimates related to *ADH1C* (Figure 2B), whereas the association between ADH1B*1/1 genotype and risk of MI became statistically significant (HR = 2.11; 95% CI: 1.02–4.05; Figure 3B). We also analyzed the association between *ADH1C* and risk of CVD stratified by alcohol consumption level (Table S1). For MI, the majority of risk estimates were not significantly different from unity. While women consuming >12 g/d and men consuming >24 g/d had a decreased risk of MI in all strata of *ADH1C*, this finding was statistically significant only for the ADH1C*1/2 genotype. In comparison, individuals belonging to the highest consumption category and carrying the slow-metabolizing ADH1C*2/2 genotype were found to be at an increased risk of stroke. Probability values for interaction between *ADH1C* genotype and alcohol consumption were 0.097 for MI and 0.074 for stroke. Because of insufficient case numbers at some indicator levels, no equivalent analyses were performed for *ADH1B*.

When pooling results from eligible prospective studies, the overall RR of CVD was 1.07 (95% CI: 0.90–1.27; p for heterogeneity 0.098) for individuals carrying the ADH1C*2/2 slow metabolizing genotype as compared to ADH1C*1/1 (Figure 2A). Subgroup analyses did not indicate differences in risk estimates between ischemic heart disease and stroke. Four publications provided HR of CVD associated with ADH1C*2/2 upon adjustment for alcohol consumption and pooling them resulted in a RR = 1.05 (95% CI: 0.83–1.32). Results from Begg's rank correlation test (p = 0.484) and Egger's (p = 0.582) indicated no significant evidence of publication bias. The funnel plot revealed a largely symmetrical distribution around the RR of 1, however, with a certain clustering around the standard error of 0.2 (Figure S1). Furthermore, the two studies with the lowest risk estimates [8], [9] and one with the largest risk estimate [7] were outside the lower 95% confidence limit. For the comparison between ADH1B*1/1 carriers and individuals with ADH1B*1/2 or *2/2*, we were unable to obtain risk estimates of one study. Thus, pooling our results with those of the only available prospective cohort study gave rise to a RR = 1.35 (95% CI: 0.98–1.88; p for heterogeneity 0.364; Figure 3A). This association became somewhat stronger after pooling risk estimates which were additionally adjusted for alcohol consumption (RR = 1.44; 95% CI: 0.99–2.08; Figure 3B).

## Discussion

In this case-cohort study nested within the EPIC-Potsdam cohort, risk of MI but not risk of stroke tended to decrease with increasing alcohol consumption. Although there was a trend towards higher alcohol consumption in individuals with the slow-metabolizing ADH1B*1/1 variant, we found no statistically significant relation between *ADH1B* or *ADH1C* genotypes and risk of cardiovascular events in our data and after pooling results of eligible prospective studies.

The J-shaped relation between alcohol consumption and risk of CHD is well established. In a meta-analysis of 28 cohort studies, RR of CHD gradually decreased up to a daily alcohol consumption of 20 g and the inverse association remained statistically significant up to 72 g/d [33]. Quite in line with the above mentioned findings, we observed a decreased risk of MI for women consuming >12 g alcohol/d and men consuming >24 g/d. Yet we had limited ability to examine MI risk associated with excessive drinking. Our finding of a ∼70% decreased risk of MI in men consuming >60 g/d was based on only seven cases and should be interpreted with caution. In women, the case numbers were quite low in any but the reference category and no female participant consuming >24 g alcohol/d suffered a MI during follow-up.

In contrast to our findings on MI, risk of stroke was not significantly related to alcohol consumption above 6 g/d. A J-shaped association between alcohol consumption and risk of stroke – most notably ischemic stroke – has been confirmed by several meta-analyses [1], [34], [35]. However, compared to CHD, the reduction in stroke risk is less pronounced and limited to lower consumption levels [1], [34]. Accordingly, our reference group is composed of individuals at low risk of stroke and therefore it is not surprising that we did not observe statistically significant risk estimates below unity. In men but not in women we also observed an increase in risk of stroke for individuals with higher alcohol consumption. Although not statistically significant, this data is in line with epidemiological evidence [1], [34], [35].

We were unable to provide risk estimates for lifelong abstainers from alcohol as only one man and 14 women of the analytical study population fell in this category. Therefore, the risk of CVD associated with zero alcohol consumption mainly reflects the group of study participants who had consumed alcohol in their past. This group may contain individuals who became abstainers from alcohol because of illness, illness-related need for medication or as they age. We tried to acknowledge this point by adjusting for prevalent diseases and by using individuals with very low alcohol consumption (>0 to 6 g/d) rather than abstainers as the reference group. The latter is justified by meta-analyses showing that former drinking is associated with an increased risk of CHD- and CVD-mortality [1], [36]. Whilst alcohol consumption of 0 g/d was not significantly related to risk of MI in our data, we observed a threefold increased risk of stroke in male abstainers from alcohol (HR = 3.57; 95% CI: 1.13–11.32). This finding even persisted after additional adjustment for lifetime alcohol consumption suggesting that changes in drinking behavior or periods of heavy drinking in the past are not the major reason why male abstainers of alcohol were at an increased stroke risk. However, risk estimates for this group were highly imprecise, as reflected by the broad 95% CI. Therefore, it is difficult to judge the strength of the association.

Potential protective effects of moderate alcohol drinking and rise in CVD risk associated with non-drinking have received a lot of attention. Although the impact of ethanol on lipoproteins, insulin sensitivity, and haemostasis provides biologically plausible pathways [2], [37], observational studies are limited in their ability to prove causality. In this respect, genetic variants associated with median alcohol consumption and/or circulating ethanol levels may act as unconfounded and precisely measured markers of the exposure and thereby contributing to strengthen causal inference [3]. In our study, a polymorphism in *ADH1B* was associated with average alcohol consumption. As expected from the higher tolerance to ethanol, individuals with the respective slow metabolizing genotype tended to consume higher amounts of alcohol than those carrying the genotype coding for the more active isoenzyme. These findings are in line with recent data showing that carriers of the rs1229984 G-allele (ADH1B*1) differed from those with the A-allele by a higher overall alcohol consumption. In this study, individuals with the G-allele even reported a higher number of drinking occasions and a higher maximum number of alcoholic drinks in a single day. Though to a lesser extent, the same tendency has been observed for rs1693482 in the *ADH1C* gene [38]. Although this SNP is in strong linkage disequilibrium with the one used in our study (rs698), we did not observe diversity in alcohol consumption across *ADH1C* genotypes. However, kinetic differences between ADH1C-isoenzymes are much less pronounced than those between ADH1B-isoenzymes [4] and therefore the impact on alcohol consumption may have been too small to be detectable with our dietary assessment instrument.

If a slower rate of ethanol clearance enhances the beneficial effect of moderate alcohol consumption on the risk of CVD, polymorphisms in ADH coding for slow ethanol metabolism should be associated with a decreased risk of CVD. Indeed, Hines et al. observed a decreased risk of MI in individuals with the ADH1C*2/2 polymorphism as compared to ADH1C*1/1 [9]. Yet, we found no such evidence in our data and after pooling the RRs of eligible prospective studies [stroke: RR = 1.15 (95% CI 0.85–1.55); ischemic heart disease: RR = 1.04 (95% CI 0.84–1.29)]. Consistent with the overall null-finding observed in prospective studies, prevalence of CVD did not differ significantly by SNPs of the *ADH1C* gene (rs1693482 and rs698) in a cross-sectional analysis of the Framingham Offspring Study [5].

To our knowledge, our study is the first investigating the association between *ADH1B* genotype and risk of stroke prospectively. Contrary to our hypothesis, we found a tendency towards an increased risk of coronary and cerebral events in carriers of the ADH1B*1/1 slow metabolizing genotype. However, only one Danish population [10] and our cohort contributed to these findings. *ADH1B* genotypes have also been assessed in a case-cohort study embedded into the prospective Danish Diet Cancer and Health Study, for which we were unable to obtain risk estimates [11]. However, the raw frequencies of *ADH1B* genotypes in cases and the random subcohort are nearly identical. Although standard methods for calculating RR and the standard error based on raw data are not applicable for the case-cohort design, the respective RR of acute coronary syndrome is likely to be close to 1. This data further supports the overall null-finding of our meta-analysis.

One may speculate that any potentially protective effect of slow ethanol oxidation might be counterbalanced by a higher tolerance to ethanol and thus a higher probability for hazardous alcohol consumption. However, adjustment for alcohol consumption did not materially change pooled risk estimates for ADH1C*2/2, whereas the association between ADH1B*1/1 and risk of CVD was even strengthened. Additionally, the impact of genotypes in ADH may only be observable in individuals with a given level of regular alcohol consumption. For the meta-analysis, we did not attempt to pool risk estimates of ADH genotypes by strata of alcohol consumption because of rather heterogeneous consumption categories - especially with regards to the reference category. Yet, previous studies analyzing this issue were rather inconsistent showing the strongest inverse association between ADH1C*2/2 genotype and risk of coronary events either in men with the highest alcohol consumption of at least one drink/d [8], [9], or in men with a modest consumption of 1–3 alcoholic beverages/week [12]. Other studies provided little evidence for an association between *ADH1C* genotype and coronary events [10], [11] or stroke [13] across strata of alcohol consumption and the same applies to the association between *ADH1B* genotype and acute coronary syndrome [11]. In our study, there was no clear evidence that the slow-metabolizing ADH1C*2/2 genotype decreases risk of CVD in any stratum of alcohol consumption (Table S1). Rather, we observed an increased risk of stroke in study participants with this genotype who exceeded the recommended upper limit of alcohol consumption of two standard drinks a day for men (24 g/d) and one standard drink a day for women (12 g/d) [24]. Although a chance finding cannot be ruled out, a slow conversion of ethanol to acetaldehyde may also amplify the well-established rise in stroke risk associated with higher consumption levels. Among abstainers from alcohol, only 16 cases of MI and 12 cases of stroke have been observed (including 1 and 3 lifelong abstainers, respectively). That is why risk estimates were highly imprecise for this group, but a tendency towards an increased risk of CVD was apparent in most strata of the *ADH1C* genotype. Therefore, the potential of negatively biasing the reference group by including former drinkers should also be acknowledged in studies investigating interactions between genetic variants with alcohol consumption.

Taken together, we observed the expected association between alcohol consumption and risk of MI or stroke, respectively. Yet, genetic variants in *ADH1B* and *ADH1C* had a minor influence on the above associations and were not significantly related to the incidence of cardiovascular events. Because of this discrepancy in findings, one may speculate that residual confounding, misreporting of alcohol consumption or constituents of alcoholic beverages other than ethanol are responsible for the well-documented J-shaped association between alcohol consumption and cardiovascular events. Given the consistency of epidemiological data across several populations with different drinking behaviors, these effects would need to be strong. Alternatively, the *in vivo* effect of the investigated genetic variants on circulating ethanol levels or alcohol drinking behaviors may not be sufficient to make causal inference about the role of alcohol consumption on the investigated outcomes. For a Mendelian Randomization approach, genetic variants of *ALDH2* with a strong influence on alcohol consumption might be more promising and have been successfully applied to study the impact of alcohol consumption on hypertension [39] and esophageal cancer [40]. On the other hand, the principal applicability of ADH gene variants has been demonstrated by recent studies showing that a slow-metabolizing genotype increases the risk of cancers of the upper aerodigestive tract [41], [42]. However, a large fraction of these cancers is believed to be attributable to alcohol consumption [43], making them a promising target to investigate genetically determined differences in ethanol metabolism. In comparison, the association between alcohol consumption and risk of CVD is less strong and not even linear. Thus, we can not exclude a causal effect of alcohol consumption on CVD risk, but the investigated genetic polymorphisms in *ADH1B* and *ADH1C* do not mirror such an effect.

Our study benefits from a well-characterized study population embedded into the EPIC-Potsdam cohort with detailed assessment of baseline and past alcohol consumption. Data were collected prospectively and follow-up proportions exceeded 90% [19]. As we used a case-cohort design, our findings are expected to be generalizable to the source population without the need to assess genotypes in the entire cohort [18]. Compared to data obtained by multiple 24-hour diet recalls, the validity of self-reported alcohol consumption in the FFQ was very good [44]. We confined our meta-analysis to prospective studies to reduce the possibility that genotypes potentially associated with the severity of disease may affect the participation rate. As only two study populations on *ADH1B* polymorphisms met our inclusion criteria, we still had limited power to detect changes in CVD risk associated with the slow-metabolizing ADH1B*1/1 genotype. Despite this limitation we believe that low statistical power can not completely explain our findings because pooled risk estimates are not in the expected direction. In addition, cross-sectional or retrospective data on this topic are also inconsistent. In a hospital-based case-control study from China, a somewhat lower prevalence of the slow-coding ADH1B*1/1 genotype was observed in patients with premature coronary artery disease (CAD) as compared to patients with late onset CAD (6.6% vs. 12.3%; p = 0.064) [45]. In comparison, male but not female carriers of the ADH1B*1 allele had a higher prevalence of cerebral and lacunal infarction in a cross-sectional Japanese study [15].

In summary, we found increasing alcohol consumption to be associated with a decreased risk of MI, whereas there was a tendency towards an increased risk of stroke. Contrary to our hypothesis, however, polymorphisms in *ADH1B* and *ADH1C* with an influence on the rate of ethanol oxidation do neither explain nor reflect the well-described impact of alcohol consumption on CVD risk.

## Supporting Information

Figure S1 **Funnel Plot for association studies of rs698 or rs1693482 in the** ***ADH1C*** **gene and CVD.** Solid vertical line represents summary risk estimate from random effects meta-analysis. Dashed lines represent 95% CI for the expected distribution of studies in absence of heterogeneity. [§] Data from the EPIC-Potsdam study [12]. Unpublished data from the Second Northwick Park Heart Study, obtained by personal communication with the corresponding author.
(TIF)

Table S1 **Relative Risks of MI and stroke across genotypes of** ***ADH1C*** **and categories of baseline alcohol consumption.** Footnote: ^§^Stratified by age at recruitment and adjusted for gender, BMI, waist circumference, smoking status, educational attainment, physical activity, non-alcohol energy intake, prevalent hypertension, prevalent diabetes mellitus, and plasma total cholesterol level. F: female participants. M: male participants.
(DOC)
