# Supplementary figures and images for: Alcohol Consumption, Genetic Variants in Alcohol Deydrogenases, and Risk of Cardiovascular Diseases: A Prospective Study and Meta-Analysis
Source: PLoS One. 2012 Feb 21;7(2):e32176. doi: 10.1371/journal.pone.0032176 (PMC3283737; doi:10.1371/journal.pone.0032176)

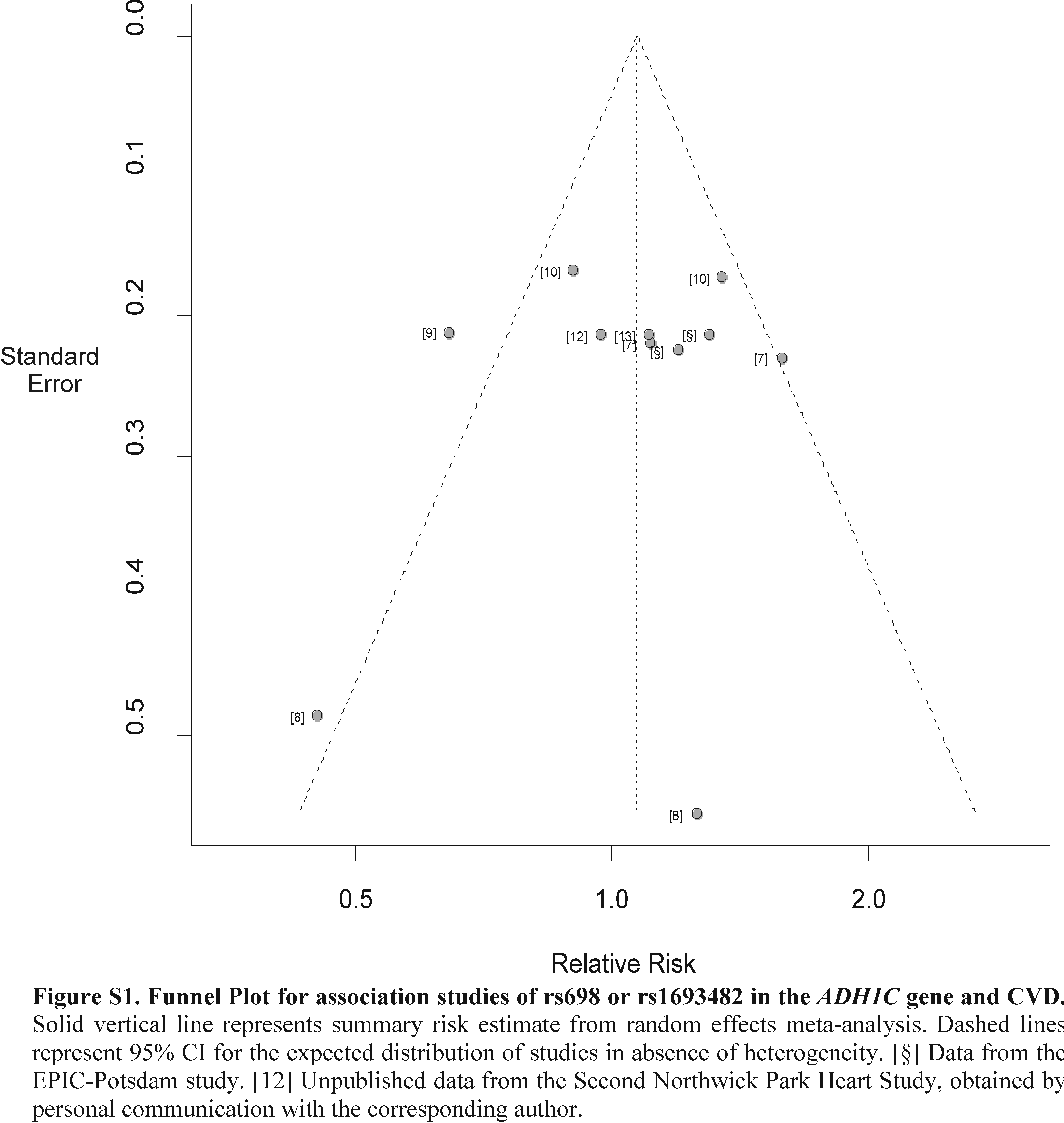

Supplement: Figure S1 — Funnel Plot for association studies of rs698 or rs1693482 in the ADH1C gene and CVD. Solid vertical line represents summary risk estimate from random effects meta-analysis. Dashed lines represent 95% CI for the expected distribution of studies in absence of heterogeneity. [§] Data from the EPIC-Potsdam study [12]. Unpublished data from the Second Northwick Park Heart Study, obtained by personal communication with the corresponding author. (TIF) [file pone.0032176.s001.tif]
