# Supplementary material for: Alcohol Consumption, Genetic Variants in Alcohol Deydrogenases, and Risk of Cardiovascular Diseases: A Prospective Study and Meta-Analysis
Source: PLoS One. 2012 Feb 21;7(2):e32176. doi: 10.1371/journal.pone.0032176 (PMC3283737; doi:10.1371/journal.pone.0032176)
Supplement: Table S1 — Relative Risks of MI and stroke across genotypes of ADH1C and categories of baseline alcohol consumption. Footnote: §Stratified by age at recruitment and adjusted for gender, BMI, waist circumference, smoking status, educational attainment, physical activity, non-alcohol energy intake, prevalent hypertension, prevalent diabetes mellitus, and plasma total cholesterol level. F: female participants. M: male participants. (DOC) [file pone.0032176.s002.doc]

Table S1. Relative Risks of MI and stroke across genotypes of *ADH1C* and categories of baseline alcohol consumption.

| **Outcome** |  | | | **0 g/d** | | | **>0 to 12 g/d (F)**  **>0 to 24 g/d (M)** | | | **>12 g/d (F)**  **>24 g/d (M)** | | |  |
| --- | --- | --- | --- | --- | --- | --- | --- | --- | --- | --- | --- | --- | --- |
| **MI** |  | | |  | | |  | | |  | | |  |
|  | Cases (n) | | | 16 | | | 158 | | | 56 | | |  |
|  | Person-Years | | | 511 | | | 12934 | | | 5093 | | |  |
|  | HR (95% CI) | | |  | | |  | | |  | | |  |
|  |  | *ADH1C^.^1/1* | 1.93 (0.69, 5.43) | | | 1 (Ref) | | | 0.61 (0.34, 1.10) | | |  | |
|  |  | *ADH1C^.^1/2* | 0.56 (0.18, 1.70) | | | 0.94 (0.64, 1.39) | | | 0.49 (0.28, 0.85) | | |  | |
|  |  | *ADH1C^.^2/2* | 4.69 (1.65, 13.35) | | | 1.11 (0.66, 1.85) | | | 0.89 (0.45, 1.76) | | |  | |
| **Stroke** |  | | |  | | |  | | |  | | |  |
|  | Cases (n) | | | 12 | | | 130 | | | 66 | | |  |
|  | Person-Years | | | 509 | | | 12804 | | | 5121 | | |  |
|  | HR (95% CI) | | |  | | |  | | |  | | |  |
|  |  | *ADH1C^.^1/1* | | | 3.28 (1.03, 10.46) | | | 1 (Ref) | | | 1.13 (0.63, 2.05) | | |
|  |  | *ADH1C^.^1/2* | | | 1.43 (0.45, 4.54) | | | 1.22 (0.81, 1.84) | | | 1.23 (0.74, 2.07) | | |
|  |  | *ADH1C^.^2/2* | | | 4.80 (1.48, 15.62) | | | 0.82 (0.45, 1.51) | | | 2.06 (1.09, 3.88) | | |

^§^ Stratified by age at recruitment and adjusted for gender, BMI, waist circumference, smoking status, educational attainment, physical activity, non-alcohol energy intake, prevalent hypertension, prevalent diabetes mellitus, and plasma total cholesterol level. F: female participants. M: male participants.
